# The South Texas Oral Health Network Study of Practitioners’ Approaches to Oral Appliance Therapy Titration for Obstructive Sleep Apnea and Their Impact on Patient Outcomes (PAOSA): Protocol for a Prospective Observational Study

**DOI:** 10.2196/96770

**Published:** 2026-06-30

**Authors:** Rahma Mungia, Ellen Funkhouser, Caitlin Sangdahl, Manju Bikkanuri, Veerasathpurush Allareddy, Frank Lobbezoo, Merel Charlotte Verhoeff, Jari Ahlberg, Gregg H Gilbert, Maria Therese Galang-Boquiren

**Affiliations:** 1 Department of Periodontics, School of Dentistry, University of Texas Health Science Center at San Antonio, 8403 Floyd Curl Drive; MC 8258, San Antonio, TX, 78229, United States, 1 5625685; 2 Division of Preventive Medicine, Department of Medicine, School of Medicine, University of Alabama at Birmingham, Birmingham, AL, United States; 3 Institute for Integration of Medicine and Science (IIMS), The University of Texas at San Antonio Health Science Center, San Antonio, TX, United States; 4 Department of Population Health Sciences, Long School of Medicine, The University of Texas at San Antonio Health Science Center, San Antonio, TX, United States; 5 Department of Orthodontics, College of Dentistry, University of Illinois Chicago, Chicago, IL, United States; 6 Department of Orofacial Pain and Dysfunction, Academic Centre for Dentistry Amsterdam (ACTA), University of Amsterdam and Vrije Universiteit Amsterdam, Amsterdam, The Netherlands; 7 Department of Orofacial Pain and Jaw Function, Faculty of Odontolog, Malmö University, Malmö, Sweden; 8 Department of Oral and Maxillofacial Diseases, Faculty of Medicine, University of Helsinki, Helsinki, Uusimaa, Finland; 9 Department of Clinical and Community Sciences, University of Alabama at Birmingham, Birmingham, AL, United States

**Keywords:** obstructive sleep apnea, oral appliance therapy, titration, dental practice-based research network, adherence, apnea-hypopnea index, patient outcomes, feasibility study, home sleep apnea testing, mean disease alleviation

## Abstract

**Background:**

Oral appliance therapy (OAT) is a widely used treatment for obstructive sleep apnea; however, titration approaches remain variable and lack standardization across clinical practice. Existing evidence is largely derived from academic or specialty sleep centers, with limited data on how titration strategies are implemented and perform in real-world dental settings.

**Objective:**

This study aims to evaluate real-world titration approaches for OAT within a dental practice-based research network and to assess their impact on treatment effectiveness and patient-centered outcomes.

**Methods:**

This prospective, multisite, observational feasibility study will enroll approximately 60 adult patients with physician-diagnosed obstructive sleep apnea receiving OAT from 10 dental practitioners within the South Texas Oral Health Network, with each practitioner enrolling up to 6 patients. Practitioners will apply either standard signs-and-symptoms–based titration or enhanced multimethod positioning titration, as per their usual clinical practice. Clinical, dental, and titration characteristics will be collected at baseline and follow-up visits over an approximately 8-week titration period. The primary outcome is mean disease alleviation, calculated as the product of physiologic efficacy (change in apnea–hypopnea index derived from multinight home sleep apnea testing) and objective adherence (hours of nightly oral appliance use captured via an embedded compliance tracker). Secondary outcomes include daytime sleepiness, sleep-related quality of life, patient satisfaction, oral appliance–related side effects, and bruxism. Descriptive analyses and generalized estimating equation models will be used to account for clustering of patients within practitioners.

**Results:**

The study was funded on June 17, 2025, and received Institutional Review Board approval from the University of Texas Health Science Center at San Antonio as the single reviewing Institutional Review Board (STUDY00001750), with site-specific approval obtained from the University of Illinois Chicago (SITE00000057). Study start-up activities, including development of data systems and coordination with device vendors, were completed between September and December 2025. Practitioner training was conducted between December 2025 and February 2026. As of March 2026, 7 dentists have been enrolled and have initiated patient recruitment. Participant recruitment began in late 2025 and is projected to continue through January 2027. Data analysis is anticipated in 2027, with study findings expected to be disseminated thereafter.

**Conclusions:**

This study will generate preliminary, real-world evidence on oral appliance titration strategies and their relationship to treatment effectiveness and patient outcomes in community dental practices. Findings will inform the design of future interventional trials and support the development of evidence-based guidance for OAT titration.

## Introduction

Obstructive sleep apnea (OSA) is a highly prevalent and underdiagnosed chronic disease characterized by repeated upper airway collapse during sleep, resulting in hypoxemia, sleep fragmentation, and sympathetic nervous system activation. Globally, OSA affects an estimated 9%‐38% of adults. In the United States, OSA is estimated to affect approximately 12% of the adult population, corresponding to nearly 54 million individuals, with up to 80% of cases remaining undiagnosed [1-4]. Untreated OSA is associated with substantial morbidity and mortality, including cardiovascular disease, metabolic dysfunction, neurocognitive impairment, motor vehicle accidents, and diminished work productivity [5]. The economic burden is profound, with undiagnosed OSA alone estimated to cost the US health care system US $149.6 billion annually [1].

Continuous positive airway pressure (CPAP) remains the first-line treatment for OSA; however, long-term adherence is poor, with fewer than half of patients maintaining adequate nightly use [6-8]. As a result, oral appliance therapy (OAT), particularly mandibular advancement devices (MADs), has become an increasingly important alternative, especially for patients with mild to moderate OSA or those intolerant of CPAP [6,8,9]. When patient adherence is considered, OAT produces clinical outcomes comparable to CPAP for many patients [7,10,11].

The therapeutic success of OAT depends on accurate titration, that is, the individualized advancement of the mandible to achieve airway patency while minimizing temporomandibular discomfort and dental side effects. The optimal therapeutic position varies substantially among patients, making titration a critical, highly individualized component of care [12]. The American Academy of Dental Sleep Medicine (AADSM) recognizes two primary approaches to titration: (1) signs-and-symptoms–based titration, relying on patient-reported outcomes such as snoring and sleepiness, and (2) multimethod positioning, which incorporates objective measures such as pulse oximetry and home sleep apnea testing (HSAT) during titration [12]. Despite widespread use of both strategies, no comparative real-world evidence exists to determine which approach optimizes outcomes, and no uniform consensus guides dental sleep medicine practice [12,13].

Traditional measures of OAT effectiveness have focused on reductions in the apnea–hypopnea index (AHI); however, AHI alone does not reflect real-world treatment benefit because adherence to therapy varies substantially across patients. Objective compliance monitoring using embedded micro trackers enables precise quantification of nightly appliance use and calculation of mean disease alleviation (MDA), which integrates both treatment efficacy (AHI reduction) and adherence [7,10]. MDA is now recognized as a superior measure of therapeutic effectiveness for OAT [7,10,14]. Advances in digital health technologies now enable the integration of objective physiologic and adherence data in routine clinical practice. The DentiTrac tracker provides objective adherence data [15], while multinight HSAT, for example, NightOwl, provides physiologic measures of sleep-disordered breathing as an alternative to polysomnography in appropriately selected patients [16-18]. Together, these tools allow for more precise and scalable evaluation of treatment effectiveness in real-world settings.

Most studies evaluating OAT titration have been conducted in academic or specialty sleep centers, limiting their generalizability to real-world dental practice. Practice-based research networks (PBRNs) offer a validated infrastructure for studying care delivery in real-world clinical environments [19-21]. The South Texas Oral Health Network (STOHN) PBRN [22,23], in collaboration with the National Dental Practice-Based Research Network (PBRN) [19-21,24], serves a highly diverse patient population, including large Hispanic, publicly insured, and uninsured communities, providing a uniquely representative setting for evaluating OSA care. Dental PBRNs facilitate the study of clinically relevant questions by integrating research into routine practice. A 2021 National Dental PBRN survey of 311 dental practitioners (general dentists and specialists) found that titration, patient compliance, and management of side effects were among the most important and least understood aspects of dental sleep medicine practice [25]. These findings highlight a critical need for practice-based, objective evidence to guide OAT titration.

The primary objective is to evaluate the feasibility and preliminary comparative effectiveness of two oral appliance therapy (OAT) titration approaches, standard signs-and-symptoms–based titration and enhanced multimethod positioning as implemented in routine clinical practice.

## Methods

### Study Design and Setting

**Figure 1.**
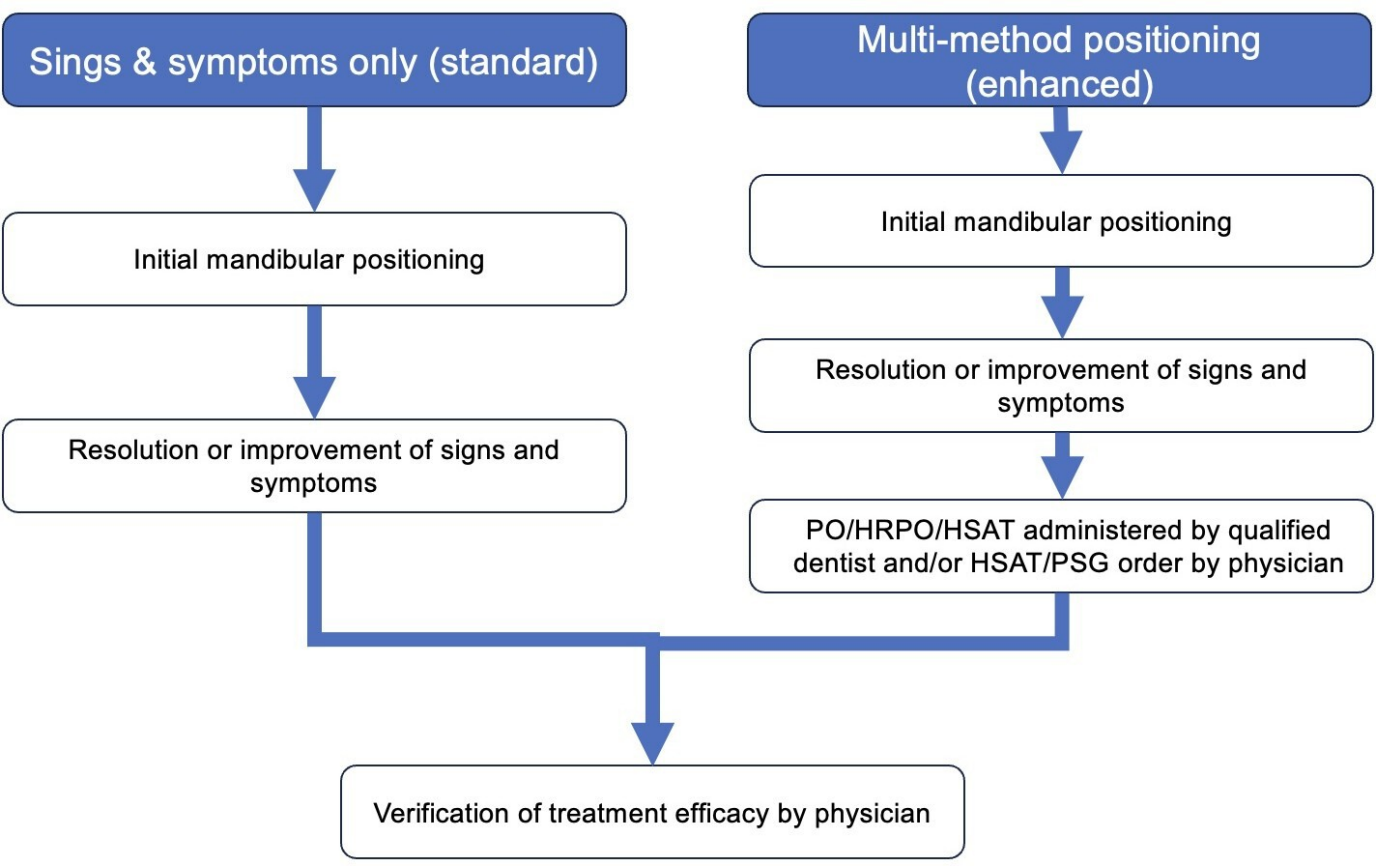
American Academy of Dental Sleep Medicine (AADSM) recommended framework for methods for positioning oral appliance. HRPO: high resolution pulse oximetry; HSAT: home sleep apnea test; PO: pulse oximetry; PSG: polysomnogram.

This is a prospective, multisite, observational feasibility study to evaluate real-world titration strategies for OAT in OSA. The study examines two titration approaches defined by the American Academy of Dental Sleep Medicine (AADSM) (Figure 1): (1) standard signs-and-symptoms–based titration and (2) enhanced multimethod positioning, which incorporates objective physiologic measures such as pulse oximetry or HSAT. Both approaches are implemented as part of routine clinical care. This study is a nonrandomized, observational investigation designed to evaluate differences in treatment effectiveness between standard signs-and-symptoms–based titration and enhanced multimethod positioning approaches. A clustered design is used, with patients nested within dentists, reflecting real-world clinical practice. Participating dentists who use both titration approaches will apply their usual method for the first 3 enrolled patients and will subsequently be asked to use the alternate approach for the remaining patients, allowing for within-practitioner comparisons. Dentists who routinely use only one titration method will continue to apply that approach consistently across all enrolled patients. For each patient, the selected titration approach is maintained throughout the titration period.

A total of 60 patients (approximately 6 per dentist) will be enrolled across 10 participating dentists over a 24-month study period, with a planned allocation favoring the enhanced titration approach to support feasibility assessment. Oral appliance therapy is delivered using MADs within routine care, with clinicians retaining discretion over device selection and design features. No protocol-driven training or mandated switching between approaches is imposed, and clinicians continue to follow their usual clinical practices. While this design enhances ecological validity, it may introduce variability related to practitioner and patient factors. These will be addressed analytically through adjustment for measured confounders and the use of generalized estimating equation models to account for clustering of patients within dentists. As a feasibility study, the primary focus is on recruitment, retention, data completeness, protocol adherence, and estimation of preliminary effect sizes to inform future interventional trials. This study protocol is reported using a structured format aligned with STROBE (Strengthening the Reporting of Observational Studies in Epidemiology) guidelines for observational studies [26].

The study is conducted within the STOHN, a regional dental practice–based research network comprising approximately 379 members across private practices, community-based clinics (eg, Federally Qualified Health Centers), and academic or faculty practice settings. Eligible practitioners must meet the following criteria: (1) be a licensed dentist (general dentist or specialist) and a current member of STOHN; (2) be qualified in dental sleep medicine (DSM), as demonstrated through prior training, continuing education, or clinical experience in managing patients with OSA; (3) have treated at least 3 patients with physician-diagnosed OSA using OAT within the past 12 months; (4) complete required human subjects protection training and study-specific training prior to patient enrollment; (5) agree to apply a defined titration approach consistently to all enrolled patients. Dentists from a range of practice settings are eligible, with no restrictions on practice type. Both general dentists and dental specialists may participate. Exclusion criteria for practitioners include participation of more than one dentist from the same practice site to minimize potential inter-dentist contamination.

Dentists from the Southwest Region of the National Dental -PBRN (n=1678) were invited to participate. In addition, the study’s principal investigators encouraged eligible colleagues within the National Dental PBRN, including those outside the Southwest Region, to participate. Eligibility criteria were consistently applied to all invited practitioners.

The study is coordinated by the University of Texas Health Science Center at San Antonio (UTHSA) Clinical Research Informatics/Data Coordinating Center and supported by Clinical and Translational Science Award infrastructure. Participating practices serve as recruitment and data collection sites. Dentist enrollment has been initiated, and patient enrollment is ongoing at the time of manuscript submission. The anticipated study duration is 24 months.

### Study Population

The study population comprises adult patients receiving OAT for OSA in participating dental practices within the STOHN. Eligible participants must meet the following criteria: (1) be aged 18 years or older; (2) have a confirmed diagnosis of OSA established by a qualified sleep physician; (3) have a referral for OAT with a scheduled oral appliance delivery visit at a participating site; (4) be able to communicate in English; (5) have access to email or text messaging for study communication; (6) be willing to complete HSAT using the NightOwl device [16,17]; (7) agree to use an oral appliance embedded with an objective compliance tracker (DentiTrac) [15].

Participants will be excluded if they: (1) lack a physician-confirmed diagnosis of OSA;

(2) have a history of OSA-related surgical treatment (eg, tonsillectomy and/or adenoidectomy or uvulopalatopharyngoplasty [UPPP]); (3) are unable to provide reliable contact information; and (4) decline participation in device-based monitoring. Participants are recruited consecutively from eligible patients receiving routine clinical care at participating sites. The titration approach is determined by the treating practitioner and applied consistently to enrolled patients within each practice.

### Recruitment and Retention

**Figure 2.**
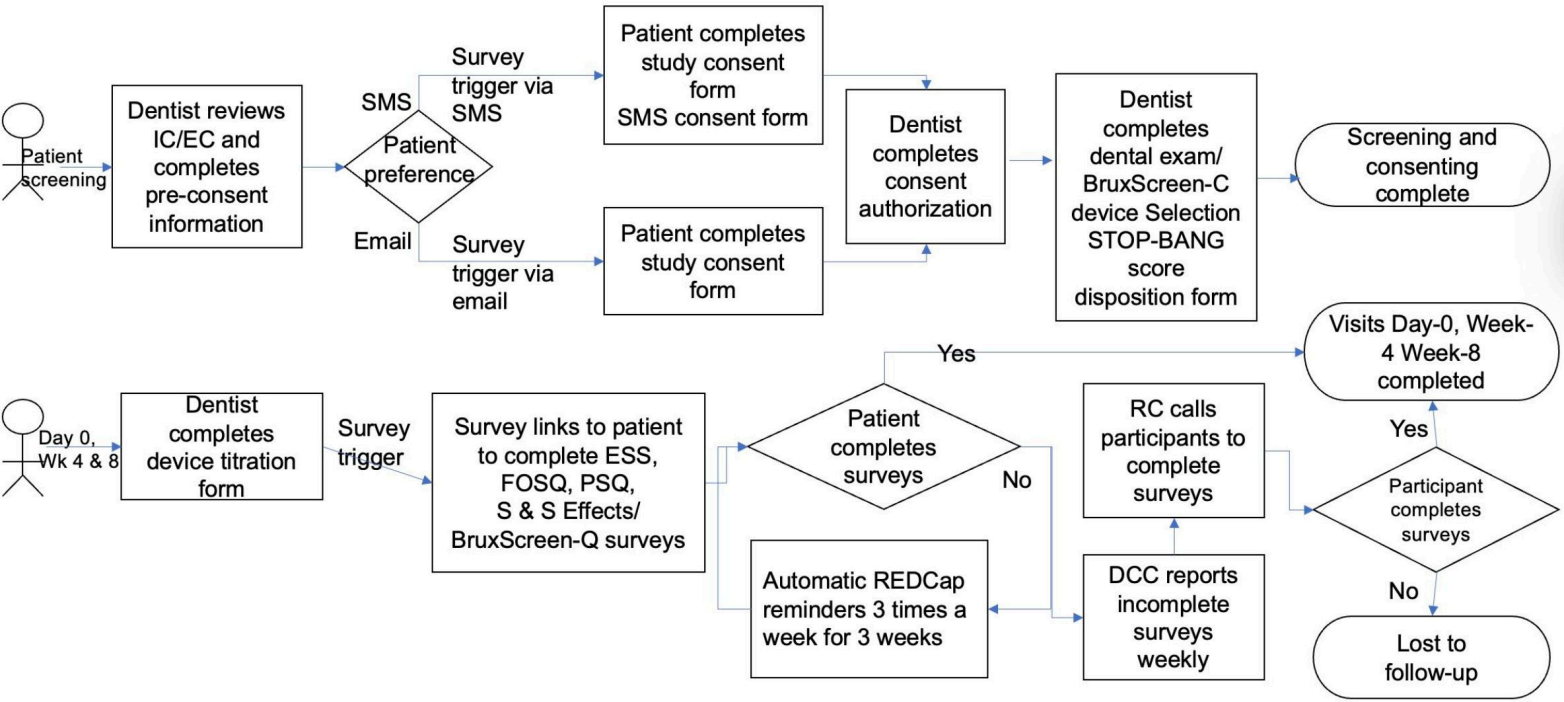
Participant survey distribution workflow. DCC: data coordinating center; EC: exclusion criteria; ESS: Epworth Sleepiness Scale; FOSQ: Functional Outcomes of Sleep Questionnaire; IC: inclusion criteria; PSQ: Patient Satisfaction Questionnaire; RC: research coordinator; REDCap: Research Electronic Data Capture.

Participating dentists will enroll eligible patients and obtain consent during routine clinical care at the time of dental examination and oral appliance prescription, with consent confirmed at the appliance delivery visit following study onboarding and training. Standardized study materials, including binders with detailed workflow diagrams (Figure 2), data collection guidance, and Research Electronic Data Capture (REDCap; Vanderbilt University) data entry forms, have been developed to support integration of study procedures into routine practice and ensure consistent data collection using REDCap, NightOwl, and DentiTrac devices. An enrollment log is maintained at each site to document screening, eligibility, enrollment, and reasons for nonparticipation.

The study team and STOHN network coordinator provide ongoing support, including protocol guidance, troubleshooting, and monitoring of recruitment progress. Dentist engagement is supported through regular communication, standardized materials, periodic recruitment updates, and continuing education opportunities. Patient retention is facilitated through reminder emails, a text messaging system (short message service, SMS), telephone follow-ups, and flexible scheduling to minimize participant burden and support continued participation.

### Study Schedule of Assessments

The study includes three study visits conducted during an approximately 8-week titration period (10 days). At visit 1a (screening and consent), eligibility is confirmed, electronic informed consent is obtained, and baseline dental examinations and clinical assessments are completed. At visit 1b (baseline), consent is confirmed, the oral appliance is delivered, baseline questionnaires are administered, and participants initiate HSAT monitoring. Follow-up assessments are conducted. at approximately 4 weeks (visit 2) and 8 weeks (visit 3). At visit 2 (4-week follow-up [10 days]), dentists perform titration adjustments as needed, collect adherence data, and have participants complete follow-up questionnaires and HSAT monitoring. At visit 3 (8-week follow-up [10 days]), final titration is performed if needed, and final adherence data, patient-reported outcomes, and HSAT assessments are collected. Following completion of study procedures, participants return to their sleep physician as part of routine clinical care.

### Data Collection Procedures and Variables

#### Overview

All study data will be collected electronically using a secure REDCap accessible via tablets, smartphones, or computers. Dentist-reported clinical data, patient-reported outcomes, and device-generated measures will be captured using standardized electronic case report forms and integrated data systems. A comprehensive summary of all variables, measurement instruments, variable roles, and data collection time points is provided in Table 1.

**Table 1.** Study variables, measures, and data collection schedule for dentists and patients.

Measurement category	Variable	Variable role^a^	Instrument and source	Time points
Dentists				
Dentist characteristics	Age, sex, years in practice, DSM^b^ training, practice type, referral patterns	Predictor	Dentist demographics form	Baseline
Eligibility	Inclusion and exclusion confirmation	Screening variable	Inclusion and exclusion criteria form	Visit^c^ 1 a
OSA^d^ risk screening	STOP-BANG score	Predictor	STOP-BANG questionnaire	Visit 1 a
Bruxism assessment	Bruxism screening (clinical indicators)	Predictor and clinical assessment	BruxScreen-C questionnaire and dental exam	Visit 1 a
Dental & airway characteristics	Occlusion, range of motion, TMJ^e^ tenderness, airway anatomy	Predictor	Dental examination form	Visit 1 a
Device selection	Appliance type, design features, rationale	Predictor	Device selection form	Visit 1b
OAT^f^ Titration Strategy	Standard versus enhanced titration	Primary Predictor (Exposure)	Device titration form	Visit 1b; Visit 2; Visit 3
Titration characteristics	Mandibular position, advancement increments, and number of adjustments	Predictor	Device titration form	Visit 1b; Visit 2; Visit 3
Clinical symptom monitoring	Snoring, fatigue, and witnessed apneas	Predictor and clinical monitoring	Dentist documentation	Visit 1b; Visit 2; Visit 3
Clinician-observed side effects	Dental discomfort, occlusal changes	Outcome (secondary)	Dentist report	Visit 2; Visit 3
Study participation	Visit completion, withdrawals, protocol deviations	Feasibility Outcome	Study tracking logs	Throughout study
Patients				
Participant characteristics	Age, sex, race and ethnicity, education, income, insurance	Predictor	Patient demographics questionnaire	Visit 1b
Bruxism assessment	Bruxism symptoms and behaviors	Secondary outcome	BruxScreen-Q questionnaire	Visit 1b
Daytime sleepiness	ESS^g^ score	Secondary outcome	Epworth Sleepiness Scale	Visit 1b; Visit 2; Visit 3
Sleep-related quality of life	FOSQ-10^h^ score	Secondary outcome	FOSQ-10 questionnaire	Visit 1b; Visit 2; Visit 3
Patient satisfaction	Satisfaction with dental sleep care	Secondary outcome	PSQ-18^i^	Visit 3
Patient-reported side effects	Jaw pain, tooth discomfort, bite changes	Secondary outcome	Sleep and Side effects questionnaire	Visit 1b; Visit 2; Visit 3
Physiologic sleep measures	AHI^j^, RDI^k^, SpO2 nadir, time <90% oxygen saturation	Primary outcome Component	NightOwlHSAT^l^	Visit 1b; Visit 2; Visit 3
Objective adherence	Hours of nightly oral appliance use; head position	Primary outcome component	DentiTrac compliance tracker	Continuous; summarized at Visit 2 and Visit 3
Treatment effectiveness	MDA^m^	Primary outcome	Derived (AHI × adherence)	Visit 2; Visit 3
Patient experience	Perceived benefit, willingness to continue therapy	Exploratory outcome	Patient questionnaire	Visit 3

a Variable roles: predictor variables represent dentist, patient, or clinical characteristics that may influence treatment outcomes. Outcome variables include patient-reported outcomes, clinician-observed effects, feasibility indicators, and the primary effectiveness outcome. Physiologic sleep measures and objective adherence data are combined to derive the primary outcome MDA, calculated as treatment efficacy (change in AHI measured by NightOwl HSAT) multiplied by objective adherence (hours of nightly oral appliance use recorded by the DentiTrac compliance micro tracker).

b DSM: dental sleep medicine.

c Visit definitions: Visit 1a = eligibility screening, consent, and baseline dental examination/clinical assessment; Visit 1b = oral appliance delivery and baseline questionnaires; Visit 2 and Visit 3 = follow-up visits during the titration period.

d OSA: obstructive sleep apnea.

e TMJ: temporomandibular joint disorder.

f OAT: oral appliance therapy.

g ESS: Epworth Sleepiness Scale.

h FOSQ: Functional Outcomes of Sleep Questionnaire.

i PSQ-18: Patient Satisfaction Questionnaire.

j AHI: apnea–hypopnea index.

k RDI: respiratory disturbance index.

l HSAT: home sleep apnea test.

m MDA: mean disease alleviation.

#### Patient-Completed Measures

Participants will complete electronic questionnaires at baseline (visit 1b), 4-week follow-up (visit 2), and 8-week follow-up (visit 3), as detailed in Table 1. These include measures of daytime sleepiness using the Epworth Sleepiness Scale (ESS) [27], sleep-related quality of life using the Functional Outcomes of Sleep Questionnaire (FOSQ-10) [28], and patient satisfaction using the Patient Satisfaction Questionnaire (PSQ-18) [29]. Participants will report oral appliance–related side effects, including jaw pain, tooth discomfort, and bite changes, at each follow-up visit. They will also undergo a bruxism screening assessment at baseline (visit 1b) using the BruxScreen-Q [30]. Participant demographic characteristics and baseline information will be collected at visit 1b.

#### Dentist-Completed Clinical Forms

Dentists will complete standardized electronic case report forms at screening (visit 1a), baseline (visit 1b), and follow-up visits (visits 2 and 3), as summarized in Table 1. These forms capture dentist and dental practice characteristics, eligibility confirmation, OSA risk screening (STOP-BANG) [31], bruxism screening using the BruxScreen-C [30], dental and airway characteristics, and oral appliance type.

#### Objective Physiologic and Adherence Measures

Physiologic sleep measures are obtained using the NightOwl HSAT device, with assessments conducted at baseline and follow-up visits as specified in Table 1. Objective adherence is measured using the DentiTrac compliance tracker embedded within the oral appliance, which records nightly usage duration and head position. Adherence data are collected continuously and summarized at follow-up visits.

#### Derived Outcomes and Data Integration

The primary outcome, MDA, is derived by integrating physiologic efficacy (change in AHI) with objective adherence, as described in Table 1. Secondary and exploratory outcomes include patient-reported measures, clinician-observed effects, and feasibility indicators. All data sources are linked to participant study identifiers within REDCap to support centralized data management and analysis.

#### Sample Size

Sixty patients will be enrolled across 10 dentist clusters (approximately 6 patients per dentist). This sample size was determined to support feasibility objectives and to estimate variability in the primary outcome (MDA) [7,10] while accounting for clustering of patients within dentists. Assuming a 2-sided α of .05 and 80% power, the study is capable of detecting standardized differences in MDA ranging from approximately 0.82 to 0.97, depending on assumptions regarding intracluster correlation coefficients (5%‐10%) and allocation ratios between titration approaches (4:6 or 3:7). These calculations account for an anticipated attrition rate of 10%‐20%.

#### Statistical Analysis Plan

Descriptive statistics will be used to summarize dentist- and patient-level characteristics, as well as primary and secondary outcomes. Continuous variables will be reported as means and standard deviations or medians and interquartile ranges, and categorical variables will be summarized using frequencies and percentages. Results will be presented for the overall cohort and stratified by titration approach. The primary analysis will evaluate differences in mean MDA between standard and enhanced titration approaches using generalized estimating equation mixed-effects models to account for clustering of patients within dentists. The titration approach will be modeled as a dichotomous independent variable. The distribution of MDA will be assessed, and transformations will be applied if necessary to meet model assumptions.

Potential confounders at the dentist and patient level will be similarly assessed, specifically, whether they are associated with the outcome, change in MDA, and with the primary independent variable, titration approach (signs and symptoms or multimethod positioning). The primary suspected and potential confounders at the dentist level will be age, practice type, and setting, and specialty. At the patient level, demographics will be similarly assessed. Typically, suspected or potential confounders would be entered in the model, and if they retained a set level of statistical significance or changed the magnitude of association between titration method and change in MDA, they would be retained as confounders. However, in this feasibility study, it is likely that the model will not converge, especially for dentist characteristics. The inability to adjust for them will be a limitation of the study and a point of discussion. Baseline OSA severity will be adjusted as a continuous variable, and depending on the distribution, may be categorized as mild, moderate, or severe. Missing data are an aspect of feasibility that will be measured and reported as such, especially for the primary variables.

### Data Management and Monitoring

**Figure 3.**
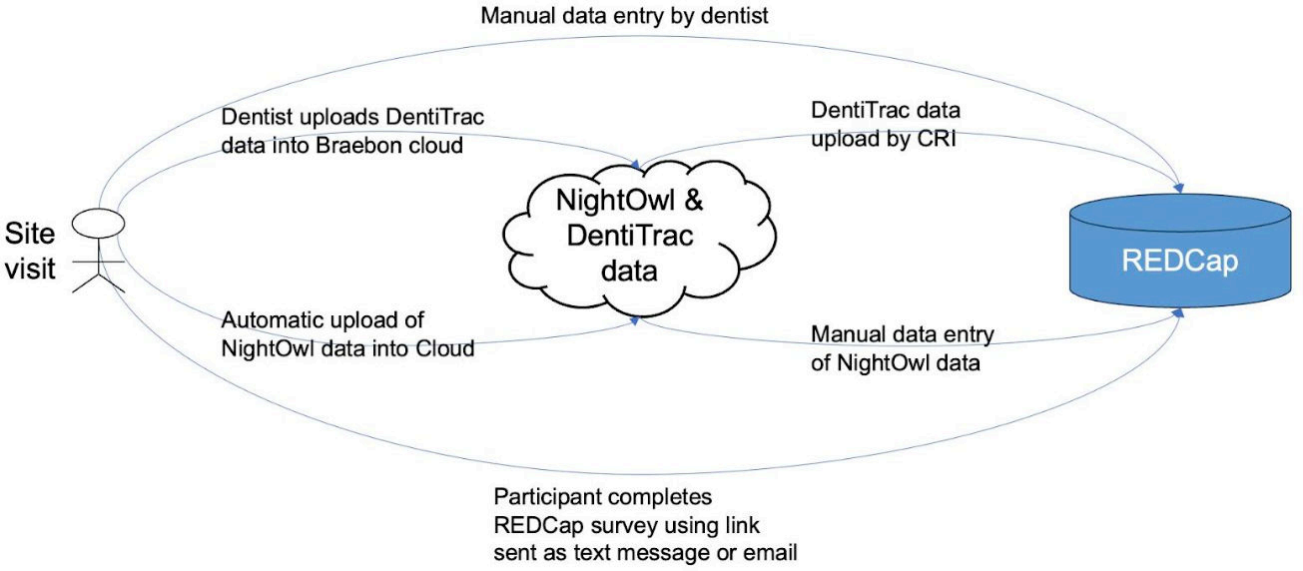
Data flow diagram. RedCap: Research Electronic Data Capture.

All study data will be entered into a secure REDCap system managed by the CRI Division at UT Health San Antonio, with built-in validation checks to ensure data quality and consistency. Dentist-reported, patient-reported, and device-generated data are integrated and linked using unique participant identifiers. As illustrated in Figure 3, data originate from multiple sources, including REDCap entry, NightOwl HSAT, and DentiTrac compliance systems, and are centralized through coordinated manual and automated data flows. Data are encrypted during transmission, stored on secure servers, deidentified for analysis, and tracked with audit trails. Ongoing data quality is maintained through centralized monitoring, routine checks for missing or inconsistent data, and resolution of discrepancies in collaboration with participating sites. Monitoring activities include remote data review and evaluation of recruitment, retention, and protocol adherence to ensure compliance with regulatory standards. This study is minimal risk. Safety monitoring focuses on unanticipated problems or adverse events related to study participation. Principal investigators conduct quarterly safety reviews, and all reportable events are submitted to the UTHSA Institutional Review Board (IRB) in accordance with institutional policy.

### Dissemination, Data Sharing, and Protocol Amendments

Study findings will be disseminated through peer-reviewed publications, presentations at national and international scientific meetings, and communication with participating practitioners through practice-based research network channels to support translation into clinical practice. Authorship will follow the International Committee of Medical Journal Editors guidelines, and participating dentists will be acknowledged as part of the study network. Deidentified data underlying study findings, along with associated documentation, will be made available following publication in accordance with UTHSA institutional policies and applicable data-sharing requirements. Data will be stored in secure repositories in accordance with regulatory and funding agency (National Institute of Dental and Craniofacial Research) guidelines. Any protocol amendments, including modifications to study procedures or early termination, will be reviewed and approved by the UTHSA IRB and documented in accordance with institutional and regulatory requirements.

### Ethical Considerations

The study will be conducted in accordance with International Council for Harmonization guidelines, the Code of Federal Regulations for the Protection of Human Subjects (45 CFR 46), and applicable National Institutes of Health policies. The IRB approval was obtained from the UTHSA as the single reviewing IRB (STUDY00001750). Site-specific approval was also obtained from the University of Illinois Chicago IRB (SITE00000057). Any protocol modifications will be submitted to the IRB for approval prior to implementation and communicated to participating sites. Electronic informed consent will be obtained at visit 1a by trained personnel. Participants will receive detailed information about study procedures, risks, and benefits, and will have the opportunity to ask questions. Confidentiality will be maintained through unique study identifiers, secure systems, and restricted data access. Dentists will receive US $80 per enrolled patient. Participants will receive US $60 total (US $20 per study visit) to compensate for time and participation. All investigators will disclose potential conflicts of interest, which will be reviewed and managed in accordance with institutional policies.

## Results

The PAOSA study was funded on June 17, 2025, and received IRB approval from the UTHSA in September 2025. Study start-up activities, including coordination with device vendors (NightOwl and DentiTrac), development of data collection systems, and preparation of training materials. Dentist recruitment and onboarding began in late 2025. Between December 2025 and March 2026, 7 dentists have completed human subjects protection and study implementation training, have been enrolled in the study, and have initiated patient recruitment. All trained dentists were cleared to begin patient recruitment following completion of study readiness activities. An additional 3 dentists are in the process of completing training.

Patient recruitment began in late 2025 during routine clinical care visits. The planned sample size is 60 patients, with approximately 6 patients per dentist. The study aims to enroll 60 patients, with approximately 6 per dentist. Recruitment is projected to continue through January 2027. Data collection includes baseline assessments and follow-up visits at approximately 4 and 8 weeks, along with integration of physiologic and adherence data from device-based systems. Final analyses are planned following completion of recruitment and follow-up, with study completion projected for June 16, 2027.

## Discussion

### Summary

This study is designed to generate feasibility data and preliminary real-world evidence comparing 2 commonly used oral appliance titration strategies and their impact on adherence-adjusted treatment effectiveness and patient-centered outcomes. Specifically, we anticipate that integrating objective physiologic monitoring into titration (enhanced multimethod positioning) may improve MDA by optimizing both treatment efficacy and adherence.

Prior studies of OAT consistently demonstrate that treatment effectiveness is shaped not only by physiologic efficacy but also by adherence, tolerability, and patient experience. Comparative effectiveness research in sleep medicine has shown that when adherence [32] is incorporated into outcome assessment, oral appliances may achieve patient-centered outcomes comparable to CPAP despite lower reductions in AHI [7,14,33,34].

Evidence evaluating oral appliance titration strategies remains limited. Existing studies have largely focused on academic or specialty sleep centers, with relatively small samples and limited integration of objective adherence data or repeated physiologic monitoring [11,13,25]. Although the AADSM recognizes both signs-and-symptoms–based titration and multimethod positioning approaches, available data do not clearly establish the comparative effectiveness of these strategies in practice-based settings [12,13]. As a result, titration practices remain heterogeneous, driven largely by clinician preference, workflow constraints, and patient tolerance rather than evidence-based guidance.

Several dentistry-focused studies provide insight into how treatment-delivery factors influence outcomes [35]. Comparative evaluations of MAD designs demonstrate that improvements in symptoms, comfort, and patient-reported outcomes may vary substantially even when reductions in respiratory indices are similar [36]. These findings suggest that physiologic response alone may not adequately capture real-world effectiveness, and that titration approach, device characteristics, and patient experience may play meaningful roles in sustained treatment benefit.

Side effects and tolerability further influence adherence and effectiveness. Prior comparisons of MAD and tongue-stabilizing devices indicate similar improvements in respiratory event indices, but markedly different acceptance rates and patient-reported outcomes [37]. In these studies, lower acceptance and higher discontinuation rates were associated with reduced quality of life gains, reinforcing the importance of integrating patient experience into treatment evaluation. Collectively, these findings support the need for outcome measures that account for both efficacy and real-world use, particularly when comparing titration strategies that may differentially affect comfort, side effects, and adherence.

International consensus guidance published in 2024 further highlights persistent uncertainty regarding optimal OAT implementation. Although multidisciplinary collaboration between sleep physicians and dental providers is strongly endorsed, consensus statements acknowledge substantial variability in titration practices and limited evidence on how titration should be operationalized across diverse clinical settings [38,39]. The absence of standardized, evidence-based titration pathways outlined in these recommendations underscores the need for practice-based research evaluating how titration is delivered in routine care, particularly outside academic environments.

Long-term dental side effects associated with MAD further emphasize the importance of titration and monitoring strategies. A recent systematic review and meta-analysis demonstrated progressive dental changes over time, including reductions in overjet and overbite and alterations in incisor inclination, while skeletal effects remained minimal [40]. These findings suggest that mandibular advancement represents cumulative exposure and that its magnitude and duration may influence dental outcomes. Objective adherence data, when interpreted alongside physiologic response, provide important context for balancing airway benefit with dental tolerability and may support more individualized titration decisions over time.

Strengths of this study include its PBRN design, integration of objective physiologic and adherence data, and evaluation of titration strategies within routine clinical workflows. The PBRN design is a major strength of this study. PBRNs provide a validated infrastructure for evaluating clinical care in real-world settings across diverse populations and practice models [19-21]. Conducted within the STOHN and in collaboration with the National Dental PBRN, PAOSA reflects the heterogeneity of real-world dental practice and enhances the external validity and applicability of study findings [25].

Several limitations should be considered. The observational, nonrandomized design introduces the potential for residual confounding and selection bias, although the structured within-practitioner approach was implemented to reduce between-practitioner variability. The relatively short follow-up period focuses on early treatment response and may not capture long-term adherence patterns or dental changes. In addition, eligibility requirements related to language and device use may limit participation among some populations. These limitations are appropriate for a feasibility-focused study and are expected to inform the design of subsequent interventional trials. As a feasibility protocol, PAOSA is designed to generate critical data on recruitment, retention, data completeness, protocol adherence, and preliminary effect-size estimates to support future randomized or pragmatic trials. The study will also identify practical barriers and facilitators to integrating HSAT and objective adherence monitoring into routine dental workflows, both of which are essential for scalability and sustainability.

Future directions include the development of randomized or hybrid effectiveness-implementation trials to more definitively evaluate titration strategies, as well as expansion to broader populations and longer follow-up periods. Study findings will be disseminated through peer-reviewed publications, presentations at scientific meetings, and communication within the dental practice–based research network to support translation into clinical practice.

### Conclusions

This study will generate feasibility data and preliminary real-world evidence on oral appliance titration strategies in routine dental practice. This study addresses a critical gap in OSA management by generating real-world, adherence-adjusted evidence on oral appliance titration across diverse dental practice settings. By integrating objective physiologic and adherence measures, the study moves beyond traditional efficacy metrics to capture clinically meaningful treatment effectiveness. The findings will provide a foundation for evidence-based titration guidance, inform the design of future interventional trials, and support more personalized, patient-centered approaches to optimizing oral appliance therapy for OSA.

## Abbreviations

AADSM: American Academy of Dental Sleep Medicine
AHI: apnea–hypopnea index
ESS: Epworth Sleepiness Scale
FOSQ-10: Functional Outcomes of Sleep Questionnaire
HSAT: home sleep apnea testing
IRB: institutional review board
MAD: mandibular advancement device
MDA: mean disease alleviation
OAT: oral appliance therapy
OSA: obstructive sleep apnea
PBRN: practice-based research network
PSQ-18: Patient Satisfaction Questionnaire-18
REDCap: research electronic data capture
STOHN: South Texas Oral Health Network
STROBE: Strengthening the Reporting of Observational Studies in Epidemiology
UTHSA: University of Texas Health Science Center at San Antonio
